# Mental health service utilization is associated with retention in care among persons living with HIV at a university-affiliated HIV clinic

**DOI:** 10.1186/s12981-018-0188-9

**Published:** 2018-01-16

**Authors:** Lauren A. Saag, Ashutosh R. Tamhane, D. Scott Batey, Michael J. Mugavero, Ellen F. Eaton

**Affiliations:** 1 Division of Epidemiology, Department of Epidemiology, Vanderbilt University, 2525 West End Ave, Suite 600, Nashville, TN 37235 USA; 2 Division of Infectious Diseases, Department of Medicine, University of Alabama at Birmingham, 845 19th St. South, Birmingham, AL 35294 USA; 3 Department of Social Work, University of Alabama at Birmingham, 900 13th Street South, Birmingham, AL 35294 USA

**Keywords:** HIV, Mental illness, Substance use, Mental health services, Retention in care

## Abstract

**Background:**

Mental health (MH) comorbidities reduce retention in care for persons living with HIV (PLWH) and are associated with poor health outcomes. Optimizing retention in primary care is vital, as poor retention is associated with delayed receipt of antiretroviral (ARV) therapy, ARV non-adherence, and poor health outcomes, including failure to suppress viral load, decreased CD4 counts, and clinically significant ARV drug resistance. We hypothesized that MH service utilization would be associated with improved retention in care for patients with HIV and MH comorbidities.

**Methods:**

This is a retrospective analysis of PLWH initiating outpatient HIV health care at a university-affiliated HIV clinic between January 2007 and December 2013. We examined the association between MH service utilization and retention in care, the outcome of interest, using univariate and multivariable logistic regression.

**Results:**

Overall, 627 (84.4%) out of 743 patients were retained in care using the Health Resources & Services Administration HIV/AIDS Bureau (HRSA/HAB) metric. A multivariable model adjusted for several sociodemographic factors, MH comorbidities, and MH service utilization. The results suggest that lack of health insurance (public ORadj = 0.3, p < 0.01; no insurance ORadj = 0.4, p < 0.01) and ≥ 3 MH comorbidities (ORadj = 0.3, P = 0.01) were associated with decreased retention in care. Conversely, older age (> 45 years, ORadj. = 1.6, p = 0.14) and ≥ 3 MH service utilization visits (ORadj. = 6.8, p < 0.01) were associated with increased retention in care.

**Conclusions:**

Even in the absence of documented MH comorbidities, improved retention in care was observed with increasing MH service utilization. In order to achieve the US-based National HIV/AIDS Strategy goal of 90% retention in care for PLWH, MH service utilization should be considered along with other evidence-based interventions to improve retention for PLWH newly engaged in care.

## Background

The goals of the US-based National HIV/AIDS Strategy (NHAS) include increasing the percentage of persons living with HIV (PLWH) who are retained in HIV primary medical care to at least 90% [1]. Currently, 56.9% of all persons ≥ 13 years with diagnosed HIV are retained in care [2]. Optimizing retention in care is vital as poor retention is associated with delayed receipt of antiretroviral (ARV) therapy, ARV non-adherence, HIV viremia, decreased CD4 counts, and drug resistance [3–7].

The “triple diagnosis” of HIV, psychiatric conditions, and substance use disorders (encompassing drug and alcohol use) complicates the management of HIV infection [8]. Mental health comorbidities (MHC), which include depression, drug use, and high-risk alcohol use [9], are common in PLWH. Major depression is over three times more prevalent in PLWH [10]. Half of PLWH report drug use [11], and rates of heavy drinking among PLWH are almost twice that of the general population [12]. Ten to 28% of PLWH have co-occurring substance use disorders and psychiatric disorders [13]. MHCs predispose PLWH to poor retention in HIV primary care (i.e. more missed visits) [14, 15] and contribute to negative health outcomes (i.e. HIV viremia, lower CD4 cell counts) [13, 16, 17]. This is not surprising given mental health disorders limit one’s ability to access health care leading to partial or no medical treatment and progressive disease.

Due to the prevalence and co-existence of MHCs in PLWH and their deleterious effects on retention in care, optimizing MHCs has the potential to foster retention in care and improve HIV-related outcomes [16, 18]. In select populations, integrating needs-based case management has been shown to increase retention in care and improve depression and drug and alcohol use [19–22] Additionally, strengths-based case management and stigma-reducing interventions [19] may ameliorate the effects of MHC on health outcomes in PLWH [23, 24]. Due to the stigma surrounding HIV, a diagnosis can be a very stressful, life-altering event [25–27] Even those without a MHC may be best managed with mental health care, such as counseling to cope with this diagnosis [25].

We hypothesized that PLWH, with and without the three examined MHCs, would experience greater retention in care if they utilized mental health services. The objective of this study is to elucidate the association between mental health (MH) service utilization (e.g. psychiatry, psychology, and substance use counseling appointments) and retention in HIV medical care. We analyzed PLWH with and without MHCs initiating HIV medical care at a US-based comprehensive clinic, which relies on federally appropriated Ryan White-safety net funds for uninsured and underinsured patients (see "Methods").

## Methods

### Study setting and population

This retrospective study was conducted at a university-affiliated HIV Clinic. This is the largest HIV outpatient center in Alabama, and it provides comprehensive medical and mental health care and case management with assistance from the Ryan White HIV/AIDS program (RWHAP). The RWHAP is federal funding that supports primary medical care and ancillary services for uninsured or underinsured PLWH [28]. The clinic maintains an electronic database including over 3200 active patients with details of clinical, socio-demographic, and psychosocial patient data. The institutional review board of the university approved this study, study forms, and protocols.

#### Eligibility criteria

Treatment naïve patients newly initiating HIV care and completing a Project CONNECT (see below) visit between January 1, 2007 and December 31, 2013 were included. Treatment naïve persons are more likely to be newly diagnosed and experiencing similar life stressors associated with HIV diagnosis and treatment. Participants were included regardless of the presence or absence of MHC. We excluded treatment-experienced persons because they have received HIV treatment and care, often including mental health services, at other clinics making them a more complex population.

### Data collection

Data was collected through two existing research protocols at the clinic. Participant data was obtained through the CFAR Network of Integrated Clinical Systems (CNICS) database, which integrates clinical data from outpatient and inpatient encounters. Clinic visit data, including the number of mental health service utilizations and primary care appointments, were obtained from CNICS to calculate exposure and outcome measures as defined below. Project CONNECT (Client-Oriented New Patient Navigation to Encourage Connection to Treatment) was initiated at the clinic in 2007 to improve linkage to medical care and has been recognized by the Centers for Disease Control and Prevention (CDC) among its compendium of best practices, and was founded on CNICS methodology, [29]. All new patients (newly-diagnosed, transferring care, or re-engaging in care after > 12 months out of care) receive Project CONNECT services including a semi-structured interview to assess a variety of sociodemographic domains (e.g., housing, HIV disclosure, transportation) and a behavioral patient-reported outcomes (PRO) questionnaire using standardized, validated instruments such as the Patient Health Questionnaire-9 (PHQ-9) [30] for depression, the Alcohol Use Disorder Identification Test-Consumption (AUDIT-C) [31] for alcohol use, and the Alcohol, Smoking and Substance Involvement Screening Test (ASSIST) [32] for substance use (alcohol use questions are excluded in ASSIST questionnaire). Project CONNECT was used as the single source for PROS. PROS are collected with touch-screen tablets or PC computers and web-based survey software developed specifically for PROs and prompt additional referrals, including mental health services from co-located psychiatry, psychology/counselor, and substance use counselors, if necessary. Data collected at the Project CONNECT visit are considered baseline and will hereafter be referred as such.

#### Outcome of interest

The outcome of interest was retention in HIV primary medical care 12 months following the Project CONNECT orientation visit. Using the Health Resources & Services Administration–HIV/AIDS Bureau (HRSA/HAB) metric [33–35] retention in care was defined as those having attended at least two primary HIV care appointments within a period of 12 months separated by at least 90 days. The number of HIV primary medical care appoints was obtained from the CNICS database.

#### Independent variables

*Mental health comorbidities:* Patients were categorized by the presence or absence of MHCs, obtained by PROs at the Project CONNECT visit. Depressive symptoms were defined by the PHQ-9 [30] and categorized into “major” (score ≥ 10) versus “mild/no” depressive symptoms (score ≤ 9) [36, 37]. The ASSIST assessed drug use and was categorized into “current” and “past/never” use [32]. We are not aware of any literature suggesting a relationship with tobacco and retention in HIV care; therefore data on tobacco usage was excluded. Because there is some evidence that marijuana may improve rather than reduce HIV treatment adherence [38], we did not include marijuana alongside other drugs which are known to negatively impact HIV outcomes. The AUDIT-C, an instrument for screening alcohol use disorders, was used to measure alcohol use in terms of “no/low risk” and “at risk” [31]. For women, a score of ≥ 3 was considered “at risk,” and < 3 was considered “no/low” risk. For men, a score of ≥ 4 was considered “at risk,” and < 4 was considered “no/low” risk. Missing data was included in the referent category, as follows: 36 (4.9%) missing depression score were categorized as “mild/no” depression, 11 (1.5%) missing drug use score were categorized as “past/never” drug use, and 13 (1.8%) missing alcohol use were categorized as “no/low risk” alcohol.

*Mental health service utilization:* MH service utilization, obtained from the CNICS database, was defined as the number of individual psychiatry, psychology, and substance use counseling visits attended in the 12 months following the Project CONNECT visit. Group therapy sessions were not included as utilization data was not available. Due to a small number of participants utilizing 4 or more MH services, MH service utilization was categorized as 0, 1, 2 or ≥ 3.

*Other variables:* Age, sex (male or female), race (white, black, other), transmission risk [heterosexual sex, intravenous drug use (IVDU), men who have sex with men (MSM), and unknown], and insurance status (public, private, uninsured) were collected from the semi-structured interview at the initial visit. The only public insurers of study participants were Medicare and Medicaid. For this analysis, insurance status is as a proxy for socio-economic status, a common practice in health services research [39–41]. Baseline CD4 cell count and HIV RNA viral load were obtained at the initial visit.

### Statistical analysis

Descriptive evaluation was performed for the total study population and by grouping participants as “retained” or “not retained” in primary care based on the HRSA-HAB definition. Continuous variables were reported as means (with standard deviations, SDs) when the distribution was normal and as medians (with quartiles, Q1 = first quartile, Q3 = third quartile) for non-normal distribution. Categorical variables were reported as frequencies (with percentages).

Associations of factors with retention in primary HIV medical care (outcome of interest) were evaluated by univariate and multivariable logistic regression. Variables were considered for inclusion into the multivariable model based on clinical relevance and statistical consideration of collinearity with other variables in the model [34]. Results are reported with unadjusted and adjusted odds ratios (OR and ORadj., respectively) and 95% confidence intervals (CIs). Age, race, sex, transmission risk factor, insurance status, number of MHCs, and number of MH service utilization visits in 12 months study period were included in the multivariable model. Model performance was examined using c-statistics, max-rescaled r-square and Hosmer–Lemeshow test for model fit. Multicollinearity of the independent factors was examined with variance inflation factor (VIF) by adjusting the linear combinations by the weight matrix used in the maximum likelihood algorithm [42]. Because the number of MHCs and each individual MHC (depression, drug use, at-risk alcohol use) were collinear, separate multivariable models were built. The first model used the ‘number of MHC’ (presented model) and the second, using each individual MH condition separately (not presented). Both models produced similar results. The VIF for all other factors was < 1.6, indicating no additional multicollinearity. While examining the effect of MHCs on the association of retention in care and MH service utilization, we encountered quasi-complete separation due to zero cell frequency. Therefore, Firth’s penalized likelihood method [43] was used to produce ORs and corresponding profile-likelihood CIs.

Statistical significance was set at 0.05 (two-tailed). All analyses were performed using SAS statistical software, version 9.3 (SAS Institute, Cary, NC).

## Results

Overall, 2797 PLWH had an initial visit between January 1, 2007 and December 31, 2013. Of these, 743 patients met inclusion criteria: treatment naïve and initiating outpatient HIV medical care. Notably, 627 (84.4%) were retained in care at 12 months. The mean age was 34.8 years (SD ± 11.3). A majority were male (82.1%), African American (61.6%), and MSM (54.8%) (Table 1). Additional patient characteristics are summarized in Table 1.

**Table 1:** Demographic and clinical characteristics of HIV-positive patients^a^ receiving care at UAB, 2007–2013 (N = 743)

Characteristic	Total	Retained^b^	Not retained^b^
	N = 743 N (col%)	N = 627 (84.4) n (row%)	N = 116 (15.6) n (row%)
Age (years), mean (SD)	34.8 ± 11.3	34.9 ± 11.4	34.2 ± 10.3
Age (years)^c^			
< 30	312 (42.0)	264 (84.6)	48 (15.4)
30–45	288 (38.8)	238 (82.6)	50 (17.4)
> 45	143 (19.2)	125 (87.4)	18 (12.6)
Race			
White	259 (34.9)	222 (85.7)	37 (14.3)
Black	458 (61.6)	385 (84.1)	73 (15.9)
Other	26 (3.5)	20 (76.9)	6 (23.1)
Sex			
Female	133 (17.9)	113 (85.0)	20 (15.0)
Male	610 (82.1)	514 (84.3)	96 (15.7)
Transmission risk			
Heterosexual	234 (31.5)	193 (82.5)	41 (17.5)
IVDU	45 (6.0)	38 (84.4)	7 (15.6)
MSM	407 (54.8)	351 (86.2)	56 (13.8)
Unknown	57 (7.7)	45 (79.0)	12 (21.0)
Insurance^c^			
Private	282 (38.0)	255 (90.4)	27 (9.6)
Public	84 (11.3)	64 (76.2)	20 (23.8)
Uninsured/unknown	377 (50.7)	308 (81.7)	69 (18.3)
CD4 count, cells/uL^c^			
< 200	244 (33.7)	211 (86.5)	33 (13.5)
≥ 200	481 (66.3)	404 (84.0)	77 (16.0)
Viral load^c^			
< 200	18 (2.5)	15 (83.3)	3 (16.7)
≥ 200	709 (97.5)	603 (85.0)	106 (15.0)
Depression^c,d^			
Yes	251 (33.7)	211 (84.1)	40 (16.0)
Mild/No	462 (61.5)	387 (83.8)	75 (16.2)
Unknown	36 (4.8)	31 (86.1)	5 (13.9)
Drug use^c,e^			
Current	129 (17.1)	103 (79.8)	26 (20.2)
Past/never	609 (81.4)	515 (81.9)	94 (15.4)
Unknown	11 (1.5)	11 (100.0)	0 (0)
At risk alcohol use^c,f,g^			
Yes	253 (34.0)	209 (82.6)	44 (17.4)
No/Low	477 (64.2)	408 (85.5)	44 (14.5)
Unknown	13 (1.7)	10 (76.9)	3 (23.1)
Number of MHCs^c,h^			
0	307 (41.3)	259 (84.4)	48 (15.6)
1	270 (36.3)	235 (87.0)	35 (13.0)
2	138 (18.6)	113 (81.9)	25 (18.1)
3	28 (3.8)	20 (71.4)	8 (28.6)
Mental healthcare utilization^i^			
0	559 (75.2)	461 (82.5)	98 (17.5)
1–2	98 (13.2)	84 (85.7)	14 (14.3)
3 +	86 (11.6)	82 (95.4)	4 (4.6)

*IVDU* intravenous drug use, *MHC* mental health co-morbidities, *MSM* men who have sex with men, *SD* standard deviation, *UAB* University of Alabama at Birmingham (Birmingham, AL, USA)

^a^Treatment naïve and newly engaged in care

^b^Definition: at least two primary care appointments being attended within a period of 12 months but separated by at least 90 days

^c^Measured at baseline (time of clinic enrollment interview)

^d^Patient Health Questionnaire-9

^e^Alcohol, smoking and substance involvement screening test

^f^Alcohol use disorder identification test-consumption

^g^Drug use excludes marijuana

^h^Measured as total count of depression, drug use, and alcohol use

^i^Measured within 12 months

Over half of patients reported ≥ one MHC based upon PRO self-assessment: 41.3% reported one MH condition, 18.6% reported two MHCs (53.6% of those with depression and alcohol use, 18.1% with depression and drug abuse, and 28.2% with alcohol use and drug abuse), and 3.8% reported all three MHCs. At-risk alcohol use was the most commonly reported MHC (34.0%), followed by “major” depressive symptoms (33.7%) and current drug use (17.1%). Despite the high burden of MHCs, three quarters (75.2%) of the sample did not have any MH service utilization: 13.2% had between one and two MH service utilizations, and 11.6% had three or more MH service utilizations during their 12-month observation period.

In Table 2, univariate and multivariable analyses examining association of retention in care with MH service utilization, MHCs, and socio-demographic variables are presented. Although none of the mental health conditions were significantly associated with retention in care in univariate analysis, an inverse dose–response relationship was observed for the number of MHCs (Table 2). In other words, as the number of MHCs increased, the odds of retention decreased. Although not statistically significant, the odds of retention for those with 3 MHC was approximately 50% lower than those without any MHC (3 MHCs = 0.5, 95% CI 0.2–1.1). Higher MH service utilization was associated with increased odds of retention in care; the association was strongest for the group utilizing ≥ 3 MH services (OR_unadj_ = 4.4; 95% CI 1.6–12.2; p = 0.01).

**Table 2:** Unadjusted and adjusted odds ratios examining association of retention^a^ in care with demographic and clinical characteristics in HIV-positive patients^b^ receiving care at UAB, 2007–2013 (N = 743)

Characteristic	Univariate analysis^c^		Multivariable analysis^c,d^	
	Unadjusted OR (95% CI)	*p*	Adjusted OR (95% CI)	*p*
Age (years)^e^				
< 30^l^	1.0	–	1.0	–
30–45	0.9 (0.6, 1.3)	0.51	0.9 (0.6, 1.4)	0.69
> 45	1.3 (0.7, 2.3)	0.43	1.6 (0.8, 3.2)	0.14
Race				
White^l^	1.0	–	1.0	–
Black	0.9 (0.6, 1.3)	0.55	1.3 (0.8, 2.2)	0.27
Other/unk	0.6 (0.2, 1.5)	0.24	0.9 (0.3, 2.5)	0.83
Sex				
Female^l^	1.0	–	1.0	–
Male	0.9 (0.6, 1.6)	0.84	0.7 (0.4, 1.4)	0.32
Transmission risk				
Heterosexual^l^	1.0	–	1.0	–
IVDU	1.2 (0.5, 2.8)	0.75	1.6 (0.6, 4.5)	0.31
MSM	1.3 (0.9, 2.1)	0.20	1.5 (0.9, 2.7)	0.13
Unknown	0.8 (0.4, 1.6)	0.54	1.1 (0.5, 2.4)	0.78
Insurance^e^				
Private^l^	1.0	–	1.0	–
Public	*0.3 (0.2, 0.6)*	*<* *0.001*	*0.3 (0.1, 0.6)*	*<* *0.001*
Uninsured/unknown	*0.5 (0.3, 0.8)*	*0.002*	*0.4 (0.3, 0.7)*	*0.001*
CD4 count, cells/uL^e^				
< 200	1.2 (0.8, 1.9)	0.38	–	–
≥ 200^l^	1.0	–		
Viral Load^e^				
< 200	0.9 (0.2, 3.1)	0.84	–	–
≥ 200^l^	1.0	–		
Depression^e,f^				
Yes	1.0 (0.6, 1.5)	0.83	–	–
No^l^	1.0	–		
Drug use^e,g^				
Current	0.7 (0.4, 1.2)	0.17	–	–
Past/never^l^	1.0	–		
At risk alcohol use^e,h,i^				
Yes	0.8 (0.5, 1.2)	0.34	–	–
No/low^l^	1.0	–		
Number of MHCs^e,j^				
0^l^	1.0	–	1.0	–
1	1.2 (0.9, 1.9)	0.36	1.2 (0.7, 1.9)	0.50
2	0.8 (0.5, 1.4)	0.51	0.7 (0.4, 1.1)	0.13
3	0.5 (0.2, 1.1)	0.09	*0.3 (0.1, 0.7)*	*0.01*
Mental healthcare utilization^k^				
0^l^	1.00		1.0	–
1–2	1.3 (0.7, 2.3)	0.43	1.6 (0.8, 3.0)	0.15
≥ 3	*4.4 (1.6, 12.2)*	*0.01*	*6.8 (2.3, 20.4)*	*<0.001*

*CI* confidence Interval, *IDU* intravenous drug use, *MHC* mental health co-morbidities, *MSM* men who have sex with men, *OR* odds Ratio, *SD* standard deviation, *UAB* University of Alabama at Birmingham (Birmingham, AL, USA)

Italic type face are statistically significant at 0.05 level

^a^Definition: at least two primary care appointments being attended within a period of 12 months but separated by at least 90 days

^b^Treatment naïve and newly engaged in care

^c^Logistic regression method

^d^Model performance: C-statistics = 0.677; Max-rescaled r-square = 9.1%; Hosmer–Lemeshow test *p* value = 0.62

^e^Measured at baseline (time of clinic enrollment interview)

^f^Patient Health Questionnaire-9

^g^Alcohol, Smoking and Substance Involvement Screening Test

^h^Alcohol Use Disorder Identification Test-Consumption Test

^i^Drug use excludes marijuana

^j^Measured as total count of depression, drug use, and alcohol use

^k^Measured within 12 months

^l^Reference category

In multivariable analyses (Table 2), persons with public insurance (OR_adj_ = 0.3; 95% CI 0.1–0.6; p < 0.001) and no insurance (OR_adj_ = 0.4; 95% CI 0.3–0.7; p = 0.001) were significantly less likely to be retained in care than privately insured PLWH. The dose response relationships for both MHCs and MH service utilization in multivariable analysis was similar to univariate analysis. There was an inverse association between 3 MHCs (OR_adj_ = 0.3; 95% CI0.1–0.7; p = 0.01) and retention in care and a positive association between ≥ 3 MH service utilization (OR_adj_ = 6.8, 95% CI 2.3, 20.4; p < 0.001) with retention in care. Both were statistically significant.

**Fig. 1:**
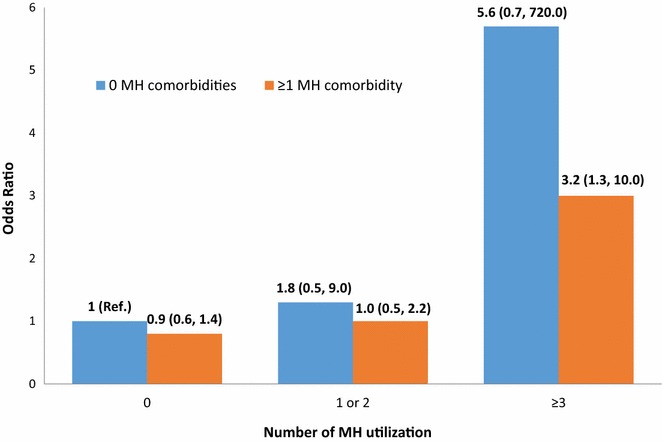
Univariate analysis of the effect of mental health comorbidities on the association of retention in care and mental health utilization in HIV-positive patients receiving care at UAB, 2007–2013 (N = 743)

An additional multivariable model (not shown) with the individual MH conditions instead of number of MHCs was examined due to collinearity between these two variables. The strength of association and statistical significance for the individual MH conditions remained the same as in univariate analyses. The results for the other predictors were similar to the model presented in Table 2. Additional analyses were completed to examine whether presence of any MH comorbidity(s) had an effect on the association between MH service utilization and retention in HIV primary care (Table 3, Fig. 1). Due to large variability and wide confidence intervals produced by Firth’s correction method, only point estimates are graphed with 95% CI labelled in Fig. 1. Among those without MH service utilization, those with ≥ 1 MHCs had reduced odds of retention (OR = 0.9; 95% CI 0.6–1.4; p = 0.74), which did not meet statistical significance. Of those with zero MHCs, odds of retention in care increased as MH service utilization increased from 0 to 1–2 and ≥ 3 service utilization visits. These associations were stronger in those without MHCs as compared to those with MHCs (1–2 MH utilization, OR = 1.8, 95% CI 0.5–9.0 vs OR = 1.0, 95% CI 0.5–2.2 and ≥ 3 MH service utilization, OR = 5.6, 95% CI 0.7–720 vs. OR = 3.2, 95% CI 1.3–10.0). In the absence of MHCs, the effect of MH service utilization on retention was increased. These relationships remained the same even when adjusted for age, race, sex, and transmission risk (data not shown on Figure).

**Table 3:** Effect of mental health comorbidities^a^ on the association of retention^b^ in care and mental health utilization in HIV-positive patients^c^ receiving care at UAB, 2007–2013 (N = 743)

Mental health utilization	Univariate analysis^d^		Multivariable analysis^d,e^	
	Mental health comorbidity		Mental health comorbidity	
	0	≥ 1	0	≥ 1
0	N = 271	N = 288		
	OR = 1.0	OR = 0.9	OR = 1.0	OR = 1.0
		(95% CI: 0.6, 1.4)		(95% CI: 0.6, 1.5)
		P = 0.74		P = 0.96
	(Reference category)		(Reference category)	
1–2	N = 23	N = 75		
	OR = 1.8	OR = 1.0	OR = 2.3	OR = 1.2
	(95% CI 0.5, 9.0)	(95% CI 0.5, 2.2)	(95% CI 0.7, 11.8)	(95% CI 0.6, 2.4)
	P = 0.37	P = 0.89	P = 0.19	P = 0.69
≥ 3	N = 13	N = 73		
	OR = 5.6	OR = 3.2	OR = 5.7	OR = 3.8
	(95% CI 0.7, 720.0)	(95% CI 1.3, 10.0)	(95% CI 0.7, 738.8)	(95% CI 1.5, 12.2)
	P = 0.12	P = 0.01	P = 0.12	P = 0.005

*CI* confidence interval, *OR* odds ratio, *UAB* University of Alabama at Birmingham (Birmingham, AL, USA)

^a^Measured as total count of depression, drug use, and alcohol use

^b^Definition: at least two primary care appointments being attended within a period of 12 months but separated by at least 90 days

^c^Treatment naïve and newly engaged in care

^d^Logistic regression method with Firth’s bias correction

^e^Adjusted for age, race, sex, and transmission risk

## Discussion

This study evaluated the association between MH service utilization and retention in HIV medical care and found a positive dose–response relationship: as utilization of mental health services increased, retention in care improved. Patients using ≥ 1mental health service were more likely to be retained in primary care at 12 months relative to those who did not. PLWH receive the greatest benefit when utilizing ≥ 3 mental health services during their first year of care regardless of the presence or absence of self-reported MHCs (depressive symptoms, drug use, and/or at-risk alcohol use, see "Methods"), which are often comorbid with HIV.

There are several explanations for the dose–response relationship between MH service utilization and retention in care. It is known that effective MHC treatment requires attention to the complex relationship between HIV and MHCs [8, 44], and integrated treatment approaches have been proven to improve the care of both HIV and MHCs [45, 46]. Additionally, contact with mental health professionals may reinforce the importance of HIV care [47]. When attending mental health appointments, patients may receive more reminders about HIV clinic appointments. For example, in our clinic, upcoming appointment reminders are printed for patients at each visit. Regardless, it is clear that exposure to more mental health services has a positive effect on clinic retention in our study both for PLWH with and without the three aforementioned MHCs. It is also noteworthy that our study evaluated PLWH newly initiating outpatient HIV medical care, a group that can be overwhelmed with their diagnosis and may benefit from case management and/or counseling [25]. Even in the absence of MHCs, contact with a mental health specialist may prompt referrals for additional support services (e.g., food supplements, travel vouchers) which may have eased the transition and attenuated the challenges often faced among persons establishing HIV medical care [34].

Our population has a high prevalence of MHCs with about one-third reporting depressive symptoms and slightly more than one-third reporting high risk alcohol use. However, a minority of patients (24.8%) received MH services during the first year of care. This is surprising as providers, social workers, and case managers in our clinic have the option to refer patients who report MHCs for mental health care at the time of PRO assessment. It is possible that the disconnect between the high prevalence of MHCs and relatively low MH utilization is due to failure to attend mental health appointments [48]. This analysis evaluated mental health utilization (visits attended) but did not include referral patterns, which would include missed appointments, cancelled appointments, or mental health services accessed outside our clinic network. Ongoing research is evaluating referral for mental health services outside of our healthcare system. We expect MH referral outside of our system is unlikely due to the comprehensive MH services provided on site in our clinic. Preliminary findings suggest that a majority of patients receiving mental health care (75%) report receiving these services on site (B. Pence, personal communication, June 26, 2017). However, the 25% who receive care elsewhere would not be captured in this study.

Notably, those with no MHCs were almost six times more likely to be retained in care if they had three or more MH utilizations in the first year. Perhaps this speaks to the prevalence of other MHCs (anxiety, schizophrenia) or the presence of unmet needs (food and/or housing) which are common in PLWH. Neither additional psychiatric conditions and unmet needs were measured in this analysis but may improve with support and resources provided through increased use of health care services, leading to improved retention in care [47, 49]. Furthermore, additional encounters with the clinic, be it for mental health care or other ancillary services, may lead to subsequent referrals for supportive care and resources. Meeting these additional needs related to food, housing, and/or transportation increases retention in HIV care [34]. By increasing the number of supportive services offered, each mental health encounter may indirectly increase retention in care. Alternatively, as clinicians, we observe the profound effect that an HIV diagnosis has on mental health and, therefore, suspect that counseling may benefit all PLWH [25–27].

This study is a moderate sized, single site assessment of MH utilization and HIV retention in care and is limited by 12-month follow-up time. Only three MHC were included (depressive symptoms, at-risk alcohol use, and drug use) and defined via self-report at the time of entry to care, which may exclude additional psychiatric conditions that may respond to MH services. Our clinic employs additional interventions including cognitive-behavioral therapy (CBT), intensive case management, and support group referrals which we were unable to query and/or measure during the study period. Study participants with MHC may have received these unmeasured services, which would potentially increase important outcomes (e.g., retention in care) due to these services rather than MH service utilization as defined by this study.

## Conclusions

Improving retention in HIV care, adherence to ARV therapies, and achieving sustained viral load suppression is critical to halting the HIV epidemic. For many PLWH with MHCs, specifically depression, drug use, and alcohol abuse, these benchmarks may only be reached with a multidisciplinary care team including mental health care. Our results suggest that MH care is important in achieving improved retention in care for PLWH, even in the absence of a patient-reported indication. Understanding the specific types and frequencies of MH services that are most likely to improve retention in care is an essential next step to the provision of integrated, comprehensive evidence-based HIV mental health care.
